# Exploring Reproductive Health Decision Experiences and Preferences of Women With Pediatric-Onset Inflammatory Bowel Diseases

**DOI:** 10.1093/crocol/otab083

**Published:** 2021-12-29

**Authors:** Zach Picciarelli, Olivia M Stransky, Mary M Leech, Hilary K Michel, Marc Schwartz, Sandra C Kim, Whitney M Gray, Traci M Kazmerski

**Affiliations:** 1 Department of Pediatrics, University of Pittsburgh School of Medicine, Pittsburgh, Pennsylvania, USA; 2 Center for Women’s Health Research and Innovation, University of Pittsburgh, Pittsburgh, Pennsylvania, USA; 3 Department of Pediatrics, Geisel School of Medicine at Dartmouth, Hanover, New Hampshire, USA; 4 Division of Pediatric Gastroenterology, Hepatology, and Nutrition, Nationwide Children’s Hospital and The Ohio State University College of Medicine, Columbus, Ohio, USA; 5 Department of Medicine, UPMC, Pittsburgh, Pennsylvania, USA

**Keywords:** sexual and reproductive health, inflammatory bowel disease, reproductive decision-making, qualitative research, reproduction in chronic disease

## Abstract

**Background:**

Women with inflammatory bowel diseases (IBDs), such as Crohn’s disease or ulcerative colitis, face several disease-specific concerns related to their reproductive health decisions. This study explored the reproductive health decision-making experiences and preferences of women with IBD to discover ways to improve this aspect of comprehensive care.

**Methods:**

We recruited women ages 18–44 years with IBD to participate in individual, semistructured interviews exploring their experiences and attitudes toward parenthood, pregnancy, contraception, and family planning care. Two independent coders performed analysis using an inductive and deductive coding approach and identified key themes.

**Results:**

Twenty-one women with IBD participated in interviews (average age 24.7 ± 5.9 years, range 18–43 years; average age of diagnosis 14.1 ± 2.0 years). We identified 4 key themes: (1) Nulliparous women who do not currently desire pregnancy appear to lack reproductive health knowledge; (2) Women with IBD lack clarity regarding the role IBD plays in contraceptive choice; (3) Related to pregnancy, women are concerned about the heredity of IBD, antepartum disease activity, and the safety of their current medications; (4) Women with IBD typically default to their reproductive health provider for reproductive health care and counseling, but they expect their gastroenterologist to initiate relevant reproductive health discussions with them and to provide information in the context of their disease.

**Conclusions:**

Women have concerns about the effects of IBD on pregnancy, parenthood, and contraceptive choice; however, many have had limited or no discussion with their gastroenterologist about the topic.

## Introduction

The reproductive decisions of women with inflammatory bowel disease (IBD) are informed by a variety of considerations and are influenced by concerns related to their underlying disease. Prior studies have shown that women with IBD have fewer children than the general population, with one-fifth to one-third of women choosing not to have children at all.^1^ Importantly, these women report concerns about increased miscarriage risks, the heritability of IBD, and medication safety during pregnancy.^2^

Children born to parents with IBD have an increased risk of developing IBD themselves; if 1 parent has IBD, the risk is 2%–9%, and if both parents have IBD, it can rise as high as 36%.^3^ Women with IBD also have a higher likelihood of worse pregnancy outcomes, such as miscarriage, preeclampsia, eclampsia, placenta previa, and placental abruption.^4^ However, with appropriate preconception planning and adequate disease control in the months prior to conception, women with IBD can have healthy pregnancies and deliveries.^5^ Most IBD medications are considered safe to take during pregnancy, with the exception of thalidomide and methotrexate, which are highly teratogenic, and prednisone, which is considered to have moderate risk.^6,7^

Women with IBD also have disease-specific concerns regarding contraceptive choice due to their increased risk for venous thromboembolism (VTE).^8^ There is no research specifically studying the impact of hormonal contraception on VTE risk in women with IBD; however, people with IBD are at a baseline increased risk for VTE, especially during periods of increased disease activity.^9^ It is unknown if women are counseled on this potential risk when selecting a method of contraception.

Considering this diverse group of concerns regarding the decision whether or not to become pregnant and options for pregnancy prevention, it is important to speak directly to women with IBD to understand what factors they deem most important for family planning. Additionally, it is important to gain insight into relevant discussions with health care providers pertaining to reproductive decision-making in the context of IBD. By exploring how women with IBD acquire information to inform their family planning decisions, we may better understand how to support women with IBD considering their reproductive goals.

We conducted a qualitative study of women with IBD to explore their experiences and goals related to reproductive health care. We focused on their disease-specific concerns regarding family planning and relevant discussions with their gastroenterologists and other health care providers. With these objectives in mind, we hoped to elucidate important aspects of care that may serve as future areas of improvement to address the specific and complex reproductive health needs of this disease population.

## Materials and Methods

Women between 18 and 43 years of age with pediatric-onset IBD (ulcerative colitis [UC] or Crohn’s disease [CD]), participated in individual interviews regarding their reproductive health needs, perspectives, and experiences. We included this range of ages to gain perspectives informed by varying degrees of reproductive experience. We chose to limit our population to those diagnosed prior to age 18 years to focus on those who have made the majority of their reproductive health decisions with their IBD in mind; simultaneously, this criterion allowed us to investigate relationships with both adult and pediatric providers as they pertain to reproductive health concerns related to IBD.

We conducted interviews over the phone to optimize participant availability and to adhere to social distancing guidelines during the COVID-19 pandemic. We offered all participants a $35USD Amazon gift card for their time.

### Interviews

We consecutively recruited individual interview participants from the University of Pittsburgh Medical Center (UPMC) Children’s Hospital of Pittsburgh Inflammatory Bowel Disease Center and the Digestive Disorders Center at UPMC Shadyside between July 2019 and July 2020. Our study team approached eligible participants via a center-based IBD research registry or during their regularly scheduled clinic visit. Interested participants provided verbal informed consent and scheduled an interview time with a research coordinator. Z.T.P. and M.M.L., medical students, and O.M.S., a Master’s level research coordinator with extensive experience conducting qualitative research with women with chronic disease, conducted all of the interviews. O.M.S. trained Z.T.P. and M.M.L. in qualitative interviewing, open-ended questioning, and active listening. The interviews explored past reproductive experiences, reproductive goals, experiences obtaining and using contraception, and the roles of their IBD care team, primary care physician, and women’s health provider in their reproductive health care (Supplementary Appendix). Interviews lasted approximately 30–50 minutes and were audio-recorded and transcribed verbatim. Z.T.P., O.M.S., and M.M.L. took notes throughout the interview and after its conclusion to allow for reflections on the interaction and early identification of potential themes. T.M.K., a pediatric subspecialist and health services researcher, served as principal investigator and provided guidance throughout the data collection phase.

### Data Analysis

M.M.L. transcribed 7 interviews and a transcription firm contracted by the University of Pittsburgh transcribed the remaining 14 interviews. We had regular team meetings throughout the project facilitated by video conferencing to include off-site collaborators and adhere to social distancing. During these meetings, we debriefed about the interview content and began to identify common themes and establish a threshold for thematic saturation. We were confident we had reached thematic saturation after 20 interviews and conducted 1 additional interview as confirmation.

We utilized Dedoose software to manage our data and facilitate the coding process. This software provides an organized interface for coding lengthy transcripts and subsequent interpretation, which facilitated a more streamlined analysis.^10^ We separately analyzed the transcripts of interviews using distinct codebooks. We used an inductive coding process in which codes were developed based on the topics that were discussed by participants.^11,12^ Two coders (Z.T.P. and O.M.S.) each independently coded 5 transcripts of the interviews before developing a preliminary codebook. The study team deductively revised the codebook during the coding of subsequent transcripts. Once a final codebook was developed, the 2 coders independently recoded the transcripts, and adjudicated these code applications to consensus. Throughout the coding process, the research team met to discuss emerging themes based on the codes developed and utilized. Once coding was completed, the research team identified themes.

### Ethical Considerations

We obtained informed consent prior to all interviews. We excluded patient identifiers from each transcript to maintain patient anonymity. This study was approved by the University of Pittsburgh Institutional Review Board. H.K.M. holds the position of Associate Editor for Crohn’s and Colitis 360 and has been recused from reviewing or making decisions for the manuscript. This study has no other conflicts of interest, financial, personal, or otherwise.

## Results

### Study Participants

Twenty-one women (average age 24.7 ± 5.9 years, age range 18–43 years; average age of diagnosis 14.1 ± 2.0 years) participated in individual interviews. Among the interviewees, 5 women had been pregnant, and 16 expressed a desire to have children in the future. Detailed demographics are noted in Table 1. The wide age range, diversity in reproductive experiences, and highly variable nature of IBD with regard to disease severity and progression produced a heterogeneity of responses. Major themes and subthemes are shared below. Participant quotations are denoted by a pseudonym, age, and diagnosis.

**Table 1.** Characteristics of participants (*n* = 21).

Age in years (mean, SD)	24.7, 5.94
Age range in years	18–43
Race/ethnicity	
White	19
Black	1
Latina	1
Number of children	
None	17
One	3
Two	1
Diagnosis	
CD	15
UC	6
Current medications	
Biologic^a^	12
5-Aminosalicylate	8
Immunomodulator^b^	5
Multiple medications^c^	6
None	1
Currently uses contraceptive	
Yes^d^	13
No	8

Abbreviations: CD, Crohn’s disease; UC, ulcerative colitis.

Patients taking infliximab, adalimumab, golimumab, or vedolizumab.

Patients taking azathioprine or methotrexate.

Patients whose drug regimens include more than 1 drug class listed.

Includes oral contraceptive pill, intrauterine device, implant, or vaginal ring (excludes condoms).

*Theme 1: Nulliparous women who do not currently desire pregnancy appear to lack reproductive health knowledge.*

Six participants described either not knowing precisely how IBD affects reproductive health or simply never having thought about the topic. This was particularly evident in women who had never given birth, especially if they did not have a current desire to become pregnant. Discussing the role IBD plays in reproductive decision-making, Sarah, a 21-year-old woman with UC, said, “Um, to be honest, I don’t know because I never asked about it. I don’t know what consequences there are, or if there’s—I don’t know. I don’t know anything about it to be honest.” When asked whether IBD plays a role in her decision to get pregnant in the future, Lydia, a 20-year-old woman with CD responded, “I guess maybe it just doesn’t right now because I know I wanna have kids, but it’s not a serious plan yet that I’m gonna pursue right now. I guess I haven’t really thought about it much, but maybe a few years into the future it would be something I thought about more.”

Cynthia, a 43-year-old woman with UC who has 1 child, implicated disease activity as an influence on seeking this knowledge, referring back to her thought process before she became pregnant: “You end up taking your health for granted when you’re healthy. It’s just what I found, and so I hadn’t even thought about the risks of being pregnant and how flares are common and likely through pregnancy. I just have been so healthy; it didn’t faze me.”

Although women often displayed a lack of knowledge regarding this interplay, they also described a desire to obtain this information when it becomes relevant to their life. This desired timeliness of patient education can be applied to both contraception and pregnancy, as is suggested by the following response from Tiffany, a 21-year-old woman with CD: “I would just say like, I guess like putting in effort to make sure, like I’m on birth control, and making sure that I’m on the type of birth control that fits best with me. And like discussing, if I were to I guess get pregnant, like the different circumstances I might have. Like if Crohn’s disease would affect it at all, if I would possibly have flare-ups when I’m pregnant. I would honestly expect them to focus more on the Crohn’s disease with the pregnancy, I guess.”

*Theme 2: Women with IBD lack clarity regarding the role IBD plays in contraceptive choice.*

This broad theme refers both to a diversity in how patients view contraception in the context of IBD and to an inconsistency in their experience discussing the topic with providers. With regard to the former, some women did not think their IBD played a significant role in what contraceptive method they use, while others considered the menstrual benefits, convenience, or even general pharmacokinetics of certain methods in relation to their disease when choosing a method.

Amber, a 24-year-old woman with UC, described contraceptive choice as a matter of convenience with regard to IBD, saying, “I would say having one less thing to worry about when you’re sick, especially with a flare-up. Having a birth control that lasts long periods that you don’t have to worry about every single day I feel is important for someone like us… Like having my Depo, and having that in the back of my mind, that’s definitely nice.” Several participants echoed this emphasis on “one less thing to worry about” in their responses.

Some participants mentioned menstrual factors as part of their contraceptive decision-making. For some women, this meant choosing a method that reduced or eliminated menstrual bleeding. For women like Blair, age 19 with a diagnosis of UC, this meant avoiding methods with unwanted effects on menstruation: “Well, maybe, some forms of birth control that have symptoms, like unpredictable bleeding and stuff, I feel those can just add so much more stress to people who already have the health condition, that I feel those are probably less popular with people with IBD. At least, that’s how I felt. I wanted something that was gonna be simple and wouldn’t cause me any more stress in my life.”

While the previous 2 quotations demonstrate similar thinking with regard to the theme, it should be noted that the issue becomes more complicated when it comes to discussing it with providers. Multiple participants felt they had not been properly counseled on choosing a contraceptive method with their IBD diagnosis in mind. Alexis, age 21 with a diagnosis of CD, described a failure to discuss how IBD medication may interact with oral contraceptive pills (OCPs) when she said, “I think I should’ve been made aware when I was put on Humira that you are not allowed to be on the pill.” Though this perceived interaction between OCPs and Humira is inaccurate (there are no adverse interactions between these drugs), it is worth noting that she was prescribed adalimumab without this aspect of her reproductive goals being elicited by the provider.^13^

In addition to discussing patients’ reproductive goals in relation to their IBD management, participants described scenarios when a patient’s IBD is left out of consideration with regard to reproductive care. This communication breakdown was described by Anita, a 25-year-old woman with CD, as she explained her perception of her gynecological care: “I wanna say that whenever I talk to a gynecologist, they ask me if I have any diseases, and I say that I have Crohn’s. Nine times out of 10, it’s just something they write down in their notes and they don’t talk about, so I don’t know if it’s just because they’re uninformed on what it is. I’m sure they know what it is, but they—I just don’t really think they put a second thought into it and how I may be different.”

*Theme 3: Related to pregnancy, women are concerned about the heredity of IBD, antepartum disease activity, and the safety of their current medications.*

When asked if IBD plays a role in her decision to have children, Alexis said, “When I was younger it used to make me want to not have kids. Because when I was flaring… I think when you’re sick with CD, there’s anger there, and you don’t want someone else to experience it. But now that I’m older and I’ve been in remission, and I see that remission is a possibility, I do want to have kids.” This demonstrates the emotions many of these women reported when considering the heredity of their disease.

Reflecting on her conversations with providers about her fears of delivery complications due to her colectomy, Amber said, “In the past when I was first diagnosed, I had a doctor around 2014 who specialized in women’s care with IBD, and we’d always talked about when the time comes—because even when I was younger, after being diagnosed, I was so fearful about getting pregnant ‘cause I was worried that something would go wrong with my disease, and it would flare-up to the point where I’d end up getting hospitalized, or again, dying on the operating table—so she would tell me that the number one thing was that my pregnancy had to be planned and I would have to be in remission before I could get pregnant. If that wasn’t the case, they would be able to take care of me, but it’d be a lot easier if I was in remission before I got pregnant, so that was always a big factor.” This woman’s experience represents effective planning and communication between a patient and provider.

When asked about how her disease may affect her reproductive health and how this interaction may influence her decisions regarding motherhood, Blair discussed her concerns and lack of clarity: “I would just like to ask if it’s going to affect my fertility and if, maybe, there are extra risks that come with pregnancy that wouldn’t be there for someone who didn’t have UC. I’d just like to know, so that could just help me decide whether I would like to actually become pregnant or adopt.”

The timing of disease activity came up several times as a factor to consider when deciding whether or not to become pregnant. Women with IBD have concerns about their disease flaring up during pregnancy, and Anita conveyed this when she said, “Definitely a bad time to get pregnant when you’re in a flare-up just because that’s just—that’s a lot. When I’m in a flare-up, I can’t even drink water without having the urgency to go to the bathroom and just pass blood. It’s pretty horrific, and so for that to be combined with a pregnancy, I think that would be my ultimate nightmare.” She went on to discuss her concerns related to the safety of her medication, saying “I’ve never had one of my GI doctors discuss that issue, which, it’s been something that I’ve worried about because I do wanna eventually have kids. I’m worried about—do I stop it before I’m planning to get pregnant? Do I stop it during? How will it affect kids, the kids that I choose to have, before, after pregnancy? It’s definitely been a concern of mine.”

*Theme 4: Women with IBD typically default to their reproductive health provider for reproductive health care and counseling, but they expect their gastroenterologist to initiate relevant reproductive health discussions and provide information in the context of their disease.*

Participants often described multidisciplinary discussions related to reproductive health care and counseling. Delilah, a 32-year-old woman with 1 child and a diagnosis of UC, described such discussions as a key way for women with IBD to avoid unintended pregnancies: “I think that having a very comprehensive conversation with both an OB [obstetrician] and a gastroenterologist on what a woman can do to prevent having pregnancy and why because of the ulcerative colitis it is really important to plan it from a health standpoint.” This theme often reflected the attitude that disease-specific reproductive care requires insight from the perspectives of both of these providers; though, as part of a closed-answer question, the majority of our participants stated that they preferred this discussion to occur primarily with their reproductive health providers.

We specifically asked the participants what they expected of their gastroenterologist with regard to family planning care. Alexis described those expectations in detail, saying “I would want them to like help me plan out and see if I was even able to be on certain medications while I was pregnant. Um, if I need to stop them for a certain period of time before I could get pregnant. Um, I’d want them to like sit down and have a conversation with me about that. And then, probably, maybe more than that, just go in maybe a couple times while I’m pregnant to make sure everything’s going okay, regarding the Crohn’s side of things, not just so much the baby. And I’d probably just want them to be involved and know what’s happening and going on, yeah.” When asked who should initiate these conversations, she echoed the sentiments of many participants, stating “I think the provider, because like, I’ve been going there for years and I still get nervous sometimes.”

When asked to describe her expectations of her gastroenterologist, Anita said, “I think it’s really important that the GI doctor is familiar with their patients and so that they know how they differ from one to another, and I think if they worked closely with family planning and everything like that, it would just result in a better outcome of that patient with having a child, and it’d be super beneficial. I know, for me, my Crohn’s is super aggressive, and so for my GI doctor to know that and be familiar with that and help me through such a big change like a pregnancy would just mean a lot to me, and it would probably really change my life in the way that I bring this child up.” She went on to give her opinion on who should initiate these discussions and how that might be best accomplished with patient comfort in mind: “I think it’s something that the providers should bring up to women once they reach a certain age or maybe even once they’re 18. I think it’s an adult conversation, but also I think there’s a way that they can bring it up without just straight going into it—like, ‘When do you plan on having kids? Would you like to know what to do if this were to happen?’—like almost asking for consent to enter that conversation.”

It should be noted that while most participants said the provider should initiate these conversations, there were some women who believed it is a shared responsibility. Even these women, however, sometimes suggested the responsibility falls slightly more on the provider. Blair expressed this when she said, “I feel maybe both [the patient and provider], but the doctor should probably bring it up because a lot of the time, the doctor’s the one leading the visit. I just feel it would be easier if the doctor would bring it up first.”

## Discussion

This study explored the concerns, experiences, and questions that women with IBD have regarding reproductive decisions. Importantly, our interviews revealed a lack of knowledge about the interactions between IBD and reproductive health and, specifically, contraceptive choices for women with IBD. Women also shared a variety of pregnancy-related concerns including IBD heritability, impact of antepartum disease activity, and safety of medications during pregnancy. Finally, women with IBD voiced the desire for reproductive health counseling and care to primarily come from reproductive health providers but wanted their IBD specialist to address disease-specific reproductive health concerns.

In this study, nulliparous women, who did not currently desire pregnancy, primarily appeared to lack knowledge about reproductive health as it related to their IBD. A 2015 questionnaire found that higher IBD-specific reproductive knowledge was associated with a higher likelihood of choosing to have children.^14^ Therefore, acquisition of accurate, disease-specific reproductive education may support women with IBD in decisions regarding pregnancy and parenthood. It is possible that this finding is both a cause and consequence of the other themes found in our study. Women with IBD may lack clarity regarding the role of their disease in contraceptive choice because they simply have not taken much time to think about it. Similar findings have been reported in studies of adolescent and young adult patients with other chronic diseases as well; for example, young people with sickle cell disease and cystic fibrosis also appear to lack reproductive health knowledge despite the impacts of their disease and medications on reproductive health concerns.^15–17^

Given the complexities of IBD care, women with IBD may prioritize other disease-specific concerns in lieu of focusing on reproductive health. Symptom management and medication adherence can be time-consuming and burdensome, especially during adolescence and the process of the transition/transfer to adult care. Additionally, adolescents with IBD have higher rates of depression and anxiety than the general population, and more severe disease tends to worsen mental health even further.^18,19^ People with IBD may lack motivation to seek IBD-specific reproductive health knowledge as other aspects of their disease require attention and their reproductive goals may be considered an issue of less immediate consequence.

Importantly, participants in our study did not describe a lack of reproductive health information availability. This is in contrast to AYA women with other pediatric-onset chronic diseases, who report dissatisfaction with the amount of information available to them regarding reproductive health in the context of their disease.^20–22^ While many women in our study described preferring that the gastroenterologist initiate disease-specific reproductive health discussions, the vast majority were satisfied with the timing, content, and comfort of those conversations. Although the provider’s ability to succeed with this is essential to minimize knowledge gaps, it seems that any such gaps for patients with IBD are less related to information availability and provision and may be more related to patient-specific factors such as the disease’s impact on other aspects of health (ie, mental health and management of symptoms and medications).

Despite some lack of reproductive health knowledge, many participants in this study highlighted disease-specific concerns related to their reproductive decisions. They frequently brought up the topic of IBD heritability, primarily citing it as a factor affecting the decision to become pregnant. This concern is likely compounded by a lack of clarity regarding the genetic profile of IBD. While twin studies and genome-wide association studies (GWASs) have proven the existence of a genetic component, the polygenic nature and significant environmental component of IBD preclude inheritance from being predictable.^23^ GWASs have identified over 230 risk alleles for IBD, some associated with both CD and UC and others associated with one or the other.^24^ With such a complex genetic picture impacted by the interactions of the gut microbiome, mucosal barrier function, inappropriate immune activation, and environmental triggers, a provider’s ability to appropriately counsel about particular patients’ heritability-based risk is limited.

Participants also described concerns regarding how IBD may affect a pregnancy, as well as how pregnancy might impact their disease activity. With regard to the former, several studies have found an increase in worse pregnancy outcomes for patients with IBD compared to the general population. A study of 2377 women with CD found women with this disease to have higher rates of preterm births, newborns small for gestational age, and cesarean section deliveries.^25^ A similar study of 2637 women with UC found higher rates of preterm births, neonatal deaths, newborns small for gestational age, and cesarean section deliveries.^26^ A case–control study looking at separate pregnancies of women pre- and postdiagnosis of IBD found that preterm birth and low birth weight were more common after diagnosis.^27^ CD activity at the time of conception or during pregnancy may increase risk for worse pregnancy outcomes.^28,29^ Similarly, several studies suggest that conceiving in remission increases the likelihood of remaining in remission for one’s entire pregnancy in both UC and CD, and, conversely, conceiving during a time of disease activity increases the likelihood of experiencing active or worsening disease during pregnancy.^30,31^ For these reasons, the Crohn’s and Colitis Foundation advises against conceiving when the patient has exacerbation of their IBD. This focus on conceiving during remission came up in several of our interviews, and was often described as being of utmost importance and represents an example of effective planning and communication between the patient and provider. Accomplishing this ideal timing can give women the best outcomes in terms of their disease, their pregnancy, and the health of their child; however, such planning can be difficult.

Several participants expressed a desire to make sure the medications they were taking would be safe to take during pregnancy. This concern specifically represents a knowledge gap that could be remedied by improved reproductive health counseling. All IBD medications are safe to take during the conception period and pregnancy except for the 2 known teratogens, methotrexate and thalidomide. Women are advised to continue all other IBD medications during pregnancy to maintain disease control and avoid negative pregnancy outcomes.^32^ Aside from the 2 major teratogens, women with IBD should also know that the common IBD medication sulfasalazine depletes folic acid levels and thus should be accompanied by a folate supplement.^33,34^ Notably, the PIANO (Pregnancy in Inflammatory Bowel Disease and Neonatal Outcomes) study found that biologics, thiopurines, and therapies combining the 2 were not associated with congenital malformations, spontaneous abortions, preterm birth, low birth weight, or infections during the first year of life.^35^ Corticosteroids, while not teratogenic, should be dose minimized as much as possible during pregnancy to avoid common side effects including hyperglycemia and hypertension.^3^

Several other topics our study participants did not delve into, though common discussion points in the literature regarding IBD and sexual and reproductive health, are fertility and mode of delivery. Active IBD and a history of pelvic surgery can affect fertility. One of our study participants discussed pregnancy as it related to her prior IBD-related surgery. It is important to note here that mode of delivery (vaginal vs cesarean section) in women with IBD is largely determined by obstetric indications and shared decision-making between a woman, her gastroenterologist, and obstetrician.^7^

Many women with pediatric-onset chronic disease view their subspecialist as a primary care provider and thus rely on them for reproductive health discussions and care.^36^ Qualitative studies have demonstrated this in cystic fibrosis and congenital heart disease.^21,37^ Interestingly, our interviews suggested that, while women with IBD desire their gastroenterologist to initiate relevant reproductive health discussions with them and to provide information in the context of their disease, they default to their reproductive health provider for this care. In this sense, women with IBD want and expect their gastroenterologist to be responsible for covering important IBD-specific aspects of their reproductive health, but do not seem to view their subspecialist as filling the role of a primary care provider or reproductive health specialist.

This interpretation underpins the fundamental principle that gastroenterologists serve an essential role in the reproductive health of women with IBD. The occurrence and efficacy of these patient–provider discussions may be a major determinant in not only the decision, but also the ability, of these women to become pregnant. Given the complicated interplay between IBD and reproductive health, collaborative care models between women’s health providers and IBD specialists are needed. Concerningly, a study found that obstetricians/gynecologists lack adequate or consistent IBD-specific knowledge regarding pregnancy.^38^ Additionally, as the pathophysiological mechanisms of and treatment options for IBD continue to grow, the role of the gastroenterologist as a team member in multidisciplinary, patient-centered comprehensive care is essential. Women with other pediatric-onset chronic disease similarly report concerns that their reproductive health providers did not have an adequate understanding of their disease. This dynamic often leaves patients confused as to whether a provider lacks pertinent knowledge or is simply deeming the disease irrelevant to reproductive care. Women with chronic disease often report lapses in communication between health care providers and functioning as an intermediary for their reproductive health care.^21,39^

This study has several limitations. Although we are confident that the themes we identified in our interviews reflect broader social norms and have meaning beyond the study population, the qualitative nature of this study means that it is not generalizable. Additionally, recruitment from only 2 locations in 1 city reflects a lack of geographic diversity. A Masters’ level research coordinator and medical students conducted our individual interviews. As is true of qualitative research, the positionality of our study team, including gender, parity, and relationship status, may have influenced the interview interactions. Through debriefings and regular team meetings with experts in pediatric and adult IBD care, as well as sexual and reproductive health, we attempted to understand these influences and enhance the credibility of this study. The racial and ethnic diversity of our study is also limited, as most participants were white, and this is significant because cultural differences could be relevant to concerns and discussions about fertility and reproductive health. Despite these limitations, qualitative methods provided the ability to explore patient experiences and preferences with more depth than could be obtained via quantitative methods. Participants shared personal stories to illustrate their experiences and defend their opinions.

## Conclusion

In conclusion, women with IBD have unique concerns about the effects of their disease on pregnancy, parenthood, and contraceptive choice. Despite these concerns, many of these women have not had extensive conversations with their gastroenterologist about these topics. Many have knowledge gaps with regard to how their disease interplays with reproductive health and some report receiving conflicting advice from different providers. Given the heterogeneous nature of IBD’s presentation, it would be helpful to obtain the perspectives of a larger sample of women with this disease. Additionally, conducting similarly focused interviews with both reproductive health providers and gastroenterologists may further guide the development of interventions to improve reproductive health care in this population.

## Supplementary Material

otab083_suppl_Supplementary_Material

## Data Availability

Data (interview transcripts) not publicly available.
