# Supplementary material for: Exploring Reproductive Health Decision Experiences and Preferences of Women With Pediatric-Onset Inflammatory Bowel Diseases
Source: Crohns Colitis 360. 2021 Dec 29;4(1):otab083. doi: 10.1093/crocol/otab083 (PMC9802148; doi:10.1093/crocol/otab083)
Supplement: otab083_suppl_Supplementary_Material [file otab083_suppl_supplementary_material.docx]

**PATIENT ID NUMBER: ______________ (record prior to interview)**

*Interview Date: ____________*

*Time Interview Began: _______*

*Time Interview Ended: _______*

**Note: Verbal consent will already have been done at this point. If there is a lag in time between the verbal consent and when the interview is scheduled, the script below will be used to re-orient the participant to the study.**

**INTRODUCTION:** (OPTIONAL)

Hello. My name is XXX. I am very appreciative that you are taking the time to talk with me today. The purpose of this research study is to hear about your experiences and perspectives regarding your reproductive goals as they relate to your chronic disease. What we learn from talking with women like you will help healthcare providers improve the ways that they address these concerns for other young women with pediatric-onset chronic diseases.

I am going to use an audio recorder to tape our conversation. Our conversation will be entirely anonymous, which means that I won’t share your voice recording with anyone but our study team. We will not use your name or the name of anyone else you mention in this interview. We also will not inform your healthcare providers about anything we discuss in this interview, and they won’t know that you participated in the study.

I really want to make sure that I hear your story and that you feel comfortable sharing your experiences with me. All questions are voluntary, so you don’t have to answer any that you don’t want to. You can stop the interview at any time. If there are any comments or thoughts that you’d like to add, I’d love to hear them.

There are no risks or benefits to participating in this interview. I expect that the interview will take around 30 to 45 minutes to finish. You will receive a $50 electronic Amazon gift card for your participation in this study.

Do you have any additional questions about the information we just reviewed together?

If any questions do arise, please don’t hesitate to ask. I’ll also give you my information at the end of the interview so you can follow up with me if you wish.

Do you have any questions for me at this point?

I will turn on the recorder now.

1. **Personal Characteristics**

First, we’re going to start off with a few questions about you.

**1. How old are you?**

**2. What race or ethnicity are you?**

**3. Tell me about your inflammatory bowel disease.**

[Prompt] When were you diagnosed?

[Prompt] How often do you see a gastroenterologist for this disease?

[Prompt] Have you ever had any surgeries?

**4. Do you take any medications for this disease?**

*If yes***🡪 What medications do you take?** Has a doctor ever told you any of these medications are unsafe to take during pregnancy?

**5. Have you ever seen a women’s health specialist (an OB/GYN, adolescent medicine, etc.)?**

*If yes*🡪 **when was the last time you had an appointment? Can you estimate how often you see them?**

1. **Do you have a primary care provider or PCP?**

*If yes*🡪 **when was the last time you had an appointment? Can you estimate how often you see them?**

**ii. Thoughts about Pregnancy and Motherhood**

Thank you for sharing that information. Now, I’m going to ask you a few questions about your reproductive history. Some of these questions are quite personal, but they help me to put your experiences into context, and also help me to decide what questions to ask you while we talk.

1. **Have you ever been pregnant?**

****If ever pregnant or currently pregnant, go to page 4***

****If never pregnant, go to page 7***

Great. Now I’m going to ask you some questions about your experiences with pregnancy.

**Use this set of questions if *ever or currently* pregnant**

**1. Tell me about your pregnancy(-ies).**

[Prompt]: (If not currently pregnant) Did you have a live birth, adoption, miscarriage, still birth, or abortion?

[Prompt]: (If currently pregnant) Congratulations! How far along are you?

***Follow-up:* How did/does your IBD affect your pregnancy(-ies)?**

3. Did you talk to your subspecialist [insert provider type] or any other health provider about pregnancy?

*If yes***🡪** How did those conversations go? What did you talk about?

[Prompt] Were any of your healthcare providers a source of support to you? In what ways?

[Prompt] Are there any ways that your health care providers could have supported you better?

[Prompt] Did your doctors ever tell you not to get pregnant?

**4. Generally, what was good or bad about your pregnancy experience?**

**5. Some women plan to get pregnant at a certain time, while some women don’t plan to get pregnant. Did you plan your pregnancy/-ies?**

***If planned pregnancy****:*

A. What factors went into your decision to try to become pregnant?

[Prompt] Some women might think about how old they are, how many kids they already have, their finances, or their relationship with their partner.

***Follow-up:* Did your IBD or medications factor into your pregnancy plans at all?**

**[Prompt] Please tell me more about that.**

B. Did you share your pregnancy plans with your subspecialist before you got pregnant?

[Prompt] What were their responses? Were they supportive? Or not as supportive as you would have liked?

C. Did advice from your doctors factor into your decision to become pregnant?

[Probe] What advice in particular factored into your decision?

***If unplanned pregnancy:***

A. How did you feel when you learned that you were pregnant?

[Prompt] Were there any concerns that you had?

B. How did you make the decision whether or not to proceed with the pregnancy?

[Prompt] Were your partner or others supportive?

[Prompt] What did you end up deciding to do?

C. Did your IBD or medications factor into that decision at all? In what ways?

**6. What are your thoughts about having (more) children?**

***Follow-up:* Does your IBD or medications factor into that decision at all? In what ways?**

[Prompt]: Do you have any concerns, conflicts, or emotions about having children?

**7. Tell me what you think it means to be a mother.**

- ***Follow-up:* What do you think would be some of the plusses and minuses of being a mother?**
- ***Follow-up:* How would/Does your IBD affect being a mother at all? In what ways?**

**8. Have your feelings about having children changed over time? In what ways?**

1. **Have you ever considered becoming a mother in ways besides pregnancy? (adoption, surrogacy, fostering)**

*If yes*🡪 Tell me about that.

**10. In GENERAL, when do you think that women are ready to have a family/what prepares them?**

[Prompt]: What about an ideal age, life stage, factors that influence readiness, finances, relationship status, etc.?

(if not addressed above) For you personally, how does your IBD or medications affect that decision?

**Use this set of questions if n*ever* pregnant**

Great. Now I’m going to ask you some questions to explore your thoughts and feelings about pregnancy and motherhood a little more.

**1. What are your thoughts about having children?**

***Follow-up:* Does your IBD or medications factor into that decision at all? In what ways?**

[Prompt]: Do you have any concerns, conflicts, or emotions about having children?

***Follow-up: Have you ever had any thoughts about the heredity of your disease? Can you describe them?***

***Follow-up: Is this something you have discussed with your subspecialist? (this should apply to each answer for this question)***

**2. What do you think would be some of the plusses and minuses of being pregnant?**

***Follow-up:* Have your healthcare providers ever told you NOT to get pregnant?**

**3. Tell me what you think it means to be a mother.**

- ***Follow-up:* What do you think would be some of the plusses and minuses of being a mother?**
- ***Follow-up:* How would your IBD affect being a mother at all? In what ways?**

**4. Have your feelings about having children changed over time? In what ways?**

**5. (If not addressed above) Are you thinking about getting pregnant?**

**6. Do you feel that there is a possibility that you could become pregnant? Why or why not?**

[Prompt]: Has a healthcare provider ever told you that you are infertile or can’t get pregnant?

*If yes*🡪 Tell me about that.

1. **Have you ever considered becoming a mother in ways besides pregnancy? (adoption, surrogacy, fostering)**

*If yes*🡪 Tell me about that.

**8. In GENERAL, when do you think that women are ready to have a family/what prepares them?**

[Prompt]: What about an ideal age, life stage, factors that influence readiness, finances, relationship status, etc.?

**9. What kinds of things would factor into YOUR decision to have a family?**

[Prompt]: What about an ideal age, life stage, factors that influence readiness, finances, relationship status, etc.?

***Follow-up: Would your IBD or medications play a role at all? In what ways?***

**iii. Contraception**

Thank you for sharing those things. I truly appreciate how open you’ve been with me. Now, I want to move on to the topic of contraception, or birth control.

**1. Have you ever used contraception or birth control?**

*If no*🡪 What have your thoughts or opinions been regarding birth control?

*1a. If yes*🡪 **What methods(s) are you using/have you used?**

*1b.* **Tell me about your experiences with this/these method(s).**

***Follow-up:* What made you pick that/these method(s)?**

***Follow-up:* Did your IBD affect your choice of birth control method? In what ways?**

***Follow-up:* Did you have any problems with using this/these method(s)?**

[Prompts: method-specific]

- Pills: *Missed/late? Taking other medications along with the pill that may have made it less effective? Side effects? How long used? How did participant remember to take it daily? Perceived effectiveness?*
- Patch/ring: *Forgot to put in/insert a new one? Taking other medications? Side effects? How long used? How did you remind yourself to change patch/ring? Perceived effectiveness?*
- Condoms: *Always used (get an idea of how often used, perhaps in % form)? Put on in time? Slip or come off? Partners view on using them? Perceived effectiveness?*
- Depo Provera: *Did you remember to get your shot in time? Side effects? Perceived effectiveness?*
- Diaphragms: *Who fitted? Use of spermicide with it? Comfortable with method? How long used? Perceived effectiveness?*
- IUD: *Where did you go to have it inserted? How long have you had IUD? Have you had any problems with it? Perceived effectiveness?*
- Emergency contraception: *Do you keep a supply at home? Perceived effectiveness?*
- Other method(s): *Probe about consistency of use, perceived effectiveness, etc.*

*1c. If yes*🡪 **Tell me about your experiences getting birth control.**

[Prompt] Where do you go to get birth control?

[Prompt] Was/is it easy for you to get? Why or why not?

**2. Have you ever discussed birth control options or contraception with a healthcare provider?**

[Prompt] Who brought up the conversation?

[Prompt] Please tell me more about that conversation.

[Prompt] Who is your preferred provider to discuss contraception with?

**iv. Health information and Provider Discussions**

I would love to get a better sense of what you view as the healthcare provider’s role in helping you achieve your reproductive goals and family planning care.

1. **We’ve talked about pregnancy, motherhood, and contraception so far. Would you like to talk about any of these issues with your subspecialist?**

[Prompt] If yes, which?

[Prompt] Ideally, when would you like these conversations to occur? [Is there a certain age or point in life when your subspecialist should discuss this with you?] How often?

[Prompt] Who should bring this up? You or your provider?

[Prompt] Would you feel comfortable bringing up contraception with your subspecialist or other provider? How would you choose which provider to talk to about this?

[Prompt] Would you feel comfortable bringing up the topic of pregnancy with your subspecialist or other provider?

[Prompt] Would you feel comfortable bring up the topic of motherhood or becoming a parent with your subspecialist or other provider?

1. **What do you expect from your subspecialist regarding family planning care?**
2. **If you have questions about your reproductive goals that are specific to your IBD or medications, where do you go for information?**

[Prompt] Try to think of a specific example of when you had a question and what you did.

**4. Have you ever used a website to look up health information?**

[Prompt] What was your experience?

[Prompt] Do you like getting health information online?

**5. Have you ever looked up information about pregnancy, contraception, or other reproductive health issues related to your IBD?**

[Prompt] What was your experience?

[Prompt] Did you find what you were looking for? Were you satisfied with the information you found? Was there information that answered your questions, or was the information incomplete?

**6. Are you in any support groups, Facebook groups, or online communities that relate to your IBD?**

*If yes***🡪** Do these groups affect decisions you make? Any examples?

**7. Do you have any apps on your phone that you use to manage your health? If so, which ones?**

**8. Would you ever use an online resource, app, or webpage to help you to make family planning decisions or learn more about family planning issues related to your disease?**

[If yes] What do you think would be useful in a resource like that?

[If no] Why not?

**9. How would you prefer to hear about health information related to family planning/reproductive goals?**

[Prompt] For example, some people might like a sit-down discussion with their providers or educational pamphlets or websites.

[Prompt] Describe what you mean/tell me more.

**10. Are there any experiences or pieces of advice about pregnancy, birth control, being a parent, or anything else that you’d like to share with other women with IBD?**

[Prompt] In other words, what would you have liked to know when you were dealing with these issues?

[Prompt] Try to think of specific examples of things that have gone well or went poorly in your own life.

**11. What would you like to tell your subspecialist about your experiences in discussing your reproductive goals with your providers? How about your other health care providers?**

[Prompt] Try to think of specific example of things that went well or went poorly.

[Prompt] What about your other health care providers?

**12. Do you have any advice for your health care providers about how to make discussions about your reproductive goals more comfortable?**

**13. In your opinion, how can your subspecialist better support women such as yourself with reproductive goals decision making?**

[Prompt] What about your other health care providers?

**v. Terminology**

1. **A number of words have been used in the past to describe pregnancies, and I’m going to go through these words. I want you to tell me what you think the definition of each word is. There is no right or wrong answer. What do you think is the definition or a(n):**

| **Intended Pregnancy** | **Planned Pregnancy** | **Wanted Pregnancy** |
| --- | --- | --- |
| **Unintended Pregnancy** | **Unplanned Pregnancy** | **Unwanted Pregnancy** |

**2. Do you think that it is common for women to have an unintended, unplanned, or unwanted pregnancy? Why or why not?**

***Follow-up:* Do you think it is common for women with IBD to have an unintended, unplanned, or unwanted pregnancy? Do you think it is more or less common than it is for women in the general population? Why or why not?**

**3. What can be done to help women with IBD avoid an unintended, unplanned, or unwanted pregnancy?**

**vi. Reaction to Interview**

We’re done now! Thank you so much for your time today.

**Do you have any feedback for me about your experience talking with me?**

[Prompt] Is there anything else you wish I had asked about that I didn’t?

[Prompt] Is there anything I should not have asked about?

I will now turn off the audio device. Thank you very much for taking this time to talk with me. Please contact me if there you have any further questions.
